# Psychosocial support and resilience building among health workers in Sierra Leone: interrelations between coping skills, stress levels, and interpersonal relationships

**DOI:** 10.1186/1472-6963-15-S1-S3

**Published:** 2015-06-08

**Authors:** Linda Vesel, Kathryn Waller, Justine Dowden, Jean Christophe Fotso

**Affiliations:** 1 Concern Worldwide US, 355 Lexington Avenue, New York, NY 10017, USA; 2 United Nations Children’s Fund, 3 United Nations Plaza, New York, NY 10017, USA; 3 Columbia University, Mailman School of Public Health (MPH student), 722 West 168th Street, New York, NY 10032, USA

**Keywords:** stress, relationships, coping techniques, resilience, health workforce, Sierra Leone

## Abstract

**Background:**

In low- and middle-income countries, a shortage of properly trained, supervised, motivated and equitably distributed health workers often hinder the delivery of lifesaving interventions. Various health workforce bottlenecks can be addressed by tackling well-being and interpersonal relationships of health workers with their colleagues and clients. This paper uses data from the Helping Health Workers Cope (HHWC) project in a rural district of Sierra Leone to achieve three objectives. First, we describe the effect of counseling and psychosocial training on coping skills, stress levels, and provider-provider and provider-client relationships. Second, we examine whether a change in coping skills is associated with a change in relationships. Finally, we qualitatively identify key ways through which the uptake of coping skills is linked to a change in relationships.

**Methods:**

The HHWC project was implemented from February 2012 to June 2013 in Kono district in the Eastern province of Sierra Leone, with the neighboring district of Tonkolili selected as the control site. The evaluation followed a mixed-methods approach, which included a quantitative survey, in-depth interviews and focus group discussions with health workers and clients. Mean values of the variables of interest were compared across sub-populations, and correlation analyses were performed between changes in coping skills, stress levels, and changes in relationships.

**Results:**

Overall, the results demonstrate that the HHWC intervention had a positive effect on coping skills, stress levels and provider-provider and provider-client relationships. Furthermore, associations were observed between changes in coping skills and changes in relationships as well as changes in stress management skills and changes in relationships.

**Conclusions:**

Psychosocial education can have major impacts on health worker well-being and the quality of health care delivery. Integrating psychosocial counseling and training interventions into health worker pre-service and in-service curricula would allow the positive effects of the HHWC intervention to be scaled up across Sierra Leone and beyond. A roll out of the HHWC approach alongside health system strengthening initiatives could have major implications for improving health and chances of survival.

## Background

One of the main resource constraints to the delivery of lifesaving interventions and improvement of key health outcomes is the shortage of a properly trained, supervised, motivated and equitably distributed health workforce [1,2]. The global shortage of health workers currently stands at more than 3.5 million at the facility and community levels [3]. Save the Children’s Health Worker Reach Index [3] ranked Sierra Leone among the top 20 countries (144 out of 161 countries) which face the most severe shortages of health workers based on health worker density, percentage of children receiving DPT vaccine, and skilled birth attendance rate. In 2008, the country had two health workers per 10,000 population, which is significantly lower than the average of 14 health workers per 10,000 population for the African continent [3] and the World Health Organization’s (WHO) benchmark of 23 health workers per 10,000 people for providing essential health services [4].

Training, retention and placement of health workers in rural, remote areas of low- and middle-income countries (LMICs) are particularly challenging [3,5,6] and have been further exacerbated in Sierra Leone due to the civil war that occurred from 1991 to 2002 [7]. Additional health workforce barriers identified by the government of Sierra Leone include low salaries, inadequate career advancement opportunities and flaws in the management of the recruitment process [6]. Strategies are needed to increase capacity and retention of health workers [1]. In their multi-country bottleneck analysis of newborn care interventions, Dickson and colleagues [8] identified the lack of motivation of health staff to be a major health workforce barrier for seven out of eight countries surveyed with a high burden of neonatal mortality. Apart from financial incentives [8-10], factors negatively influencing motivation include heavy workload, workforce shortages, lack of inter-professional exchanges and goal-oriented supervision, and underappreciation and distrust from the community being served [11].

Various health workforce bottlenecks can be addressed by tackling well-being and relationships among health facility workers and between providers and clients [12]. Resilience, namely the ability to handle stress in a healthy and adaptive way, is a major factor in health worker well-being [13]. Stress can impact the functioning of the health system as a whole with respect to retention, performance, productivity, safety, demand and quality of care [14], and has an overall impact on health worker job satisfaction levels [15]. Teaching stress management and coping skills can prevent burnout, a condition caused by long-term, perpetual stress that leads to emotional exhaustion and one’s diminishing sense of purpose and self [16,17]. Furthermore, improved interactions among co-workers have been shown to be associated with stress reduction [14]. The WHO also defined strengthening of social relationships among health workers as a health promotion strategy linked to quality of care [12].

This paper uses data from the Helping Health Workers Cope (HHWC) project in a rural district of Sierra Leone to achieve three objectives. First, we describe the effect of counseling and psychosocial training on coping, stress, and provider-provider and provider-client relationships. Second, we examine the associations between change in coping skills, stress levels and change in relationships. Finally, we qualitatively identify key ways through which the uptake of coping skills is linked to a change in relationships. The theory of change underpinning the intervention, developed by the research team and stemming from the review of evidence and formative research, is based upon the hypothesis that by providing counseling and training on stress management, self-care and client care, health workers will gain new coping skills that in turn will lead to improved interactions amongst providers and between providers and clients. This will ultimately improve job satisfaction and motivation to deliver better services.

## Methods

### Design, setting and study population

The HHWC project was implemented from February 2012 to June 2013 in Kono district in the Eastern province of Sierra Leone, with the neighboring district of Tonkolili selected as the control site. The specific aims were to improve coping techniques among health workers by addressing workplace stressors and introducing support services, and to improve interpersonal relationships between health workers and with clients. All primary health care facilities, known as peripheral health units (PHUs), were mapped and a listing of all health workers in each facility was completed. A total of 80 PHUs and approximately 300 health workers were listed, and the project was open to every health worker employed in these facilities. A total of 271 health workers from 74 facilities in 12 out of the 14 chiefdoms in Kono district enrolled in the project. Participants included Community Health Officers, Maternal and Child Health Aides, State Registered Nurses, Vaccinators, Nursing Aides, State Enrolled Community Health Nurses and Endemic Disease Control Unit Assistants; these represented all cadres present at the PHUs.

All health workers participated in an individual intake counseling assessment, which explored their levels of stress, the specific stressors faced and their support systems. All participants took part in ten group counseling sessions held at a central location in each chiefdom. Health workers were grouped into women and men’s groups and met weekly. Those who were classified as being under severe stress or who had experienced trauma were offered additional individual counseling tailored to their needs. Following the group counseling sessions, health workers were trained on three core skill sets – stress management, self-care and client-care – each lasting one full day. Finally, refresher training was provided to all health workers after nine months to review the key lessons related to core skills.

### Data collection and analysis

The evaluation followed a mixed-methods approach, which included a quantitative survey, in-depth interviews (IDIs) and focus group discussions (FGDs). The survey data, IDIs and FGDs were collected between May and July 2013 by trained fieldworkers.

#### Quantitative survey

The quantitative component consisted of a health worker survey that was administered in the intervention and control sites. In the project district, respondents included a random sample of 129 health workers who had been engaged in the intervention, while in the comparison area, a sample of 157 health workers employed in similar cadres as the project participants was selected. The survey was administered in and around the facility premises in a place chosen to ensure privacy and comfort. To compensate for the absence of a baseline, the survey was administered after the implementation of the intervention to assess and rate both the pre- and post-intervention skills and relationships. The survey covered perceived stress, coping skills and relationships, the details of which are described below.

• **Perceived stress** related to a health worker’s psychological state around handling work, personal affairs and oneself. One aggregate variable was constructed comprising 11 individual questions answered on a four point Likert scale ranging from ‘never’ to ‘always’ (see Additional file 1).

• **Coping skills** related to health workers’ ability to build resilience as well as address interpersonal relationships at and outside of work. The aggregate variable comprised 16 questions across three sub-aggregate variables or domains – communication (four variables), self-care (five variables) and social connectedness (six variables) – answered on a four point Likert scale ranging from ‘never’ to ‘always’ (see Additional file 1).

• **Relationships** between health workers and their supervisors, co-workers at health facilities, co-workers from other health facilities nearby, traditional birth attendants working in the community, and patients visiting the facilities were self-reported. Combining the five variables described above as well as the category ‘no relationships’ created an aggregate relationship variable. The two individual variables on relationships with co-workers at health facilities, and between health workers and clients visiting health facilities were also included in the analysis. Responses were recorded on a four point Likert scale ranging from ‘good’ to ‘bad’ and the categories were re-ordered to ensure that higher values reflected better outcomes.

In the descriptive analysis, mean values of the variables on perceived stress levels, coping skills and relationships were compared across the three sub-populations, namely, the retrospective pre-test, the post-test and the control area. Chi-squared tests were used to assess the statistical significance of the differences. To test the theory of change, we assessed the extent to which improved coping skills were associated with lower levels of stress and with improved relationships. To this end, correlation analyses were performed between changes in coping skills (from pre-test to post-test), stress levels (post-test), and changes in relationships (from pre-test to post-test). In this paper, as in studies on human social behaviors, the following cut-off points were used to characterize the strength of the associations: If R ≤ 0.30, there is a weak linear relationship; if R > 0.30 and R < 0.65, there is a moderate linear relationship; and if R ≥ 0.65, there is a strong linear relationship, where R denotes the absolute value of the correlation coefficient.

#### Qualitative interviews and discussions

In-depth interviews (IDIs) were conducted with health workers in the intervention area. These were designed to gather information to complement and contextualize the findings from the survey around issues of job satisfaction, motivation, relationships and stress. The ten respondents for the IDIs were selected purposively based on project documents and consultations with project staff as well as available resources. Focus group discussions (FGDs) were also conducted with patients to elicit their views on the health services in their communities, and their interactions with health workers. A total of five FGDs were conducted in the project area, while six were conducted in the control district. Five facilities in the intervention district and six facilities in the control district were randomly selected. A catchment community from each of the 11 facilities was then selected randomly for the FGD. Each FGD included between six to ten pregnant or postpartum women recruited with the help of local community leaders.

The qualitative data was directly translated and transcribed verbatim into a Microsoft Word document, checked and manually coded by the evaluation team, and imported into the Dedoose online qualitative data analysis software. Data from each set of respondents were excerpted based on the key outcomes of interest and the emerging themes to understand any dominant issues and patterns.

### Ethical considerations

The government of Sierra Leone Ethics and Scientific Review Committee granted ethical approval for the evaluation of the project. All respondents gave verbal and written consent for the interview and audio recording of their responses. The consent forms also guarantee the anonymity and confidentiality of the responses.

## Results

### Effect of counseling on coping, stress and relationships

Table 1 presents the results of the descriptive analysis that addressed coping skills, stress levels and relationships. In the post-test period, health workers’ ability to apply coping techniques, overall and for each of the three domains (communication, self-care and social connectedness), was significantly higher in the intervention district of Kono than in the control area (p=0.000). In the intervention district, differences in coping skills from the retrospective pre-test to the post-test periods were positive and statistically significant (p=0.000). On average, health workers reported lower stress levels in the intervention district (captured by an aggregate score of 2.40) compared with the comparison district (2.48), marked by a statistically significant difference (-0.086, p=0.034).

**Table 1:** Coping skills, stress levels and relationships

	Average levels			Post-Test, Control difference		Post-Test, Pre-Test difference	
		
Variable	Post-Test (Kono)	Control (Tonkolili)	Pre-Test (Kono)				
	
	(1)	(2)	(3)	(1)-(2)	P-value	(1)-(3)	P-value
**Overall coping strategies**	3.23	2.79	2.63	0.447	0.000	0.601	0.000

Communication skills	3.42	3.05	2.26	0.367	0.000	1.156	0.000

Self-care skills	2.99	2.57	2.72	0.421	0.000	0.265	0.000

Social connectedness	3.32	2.80	2.81	0.523	0.000	0.510	0.000

**Stress levels**	2.40	2.48	NA	-0.086	0.034	NA	NA

Overall relationships^*^	3.47	3.35	2.69	0.121	0.025	0.786	0.000

With co-workers at facility	3.55	3.44	2.67	0.111	0.110	0.884	0.000

With patients	3.42	3.49	2.66	-0.072	0.320	0.764	0.000

**N**	**129**	**157**	**129**				

^*^Includes relationships with (1) co-workers at facility, (2) patients, (3) supervisors, (4) co-workers from other facilities, and (5) traditional birth attendants

Table 1 also shows that between the retrospective pre-test and post-intervention periods within Kono, there were improvements in overall relationships (p=0.000), relationships with co-workers (p=0.000), and relationships with clients (p=0.000). The difference in overall relationships between the intervention and control sites in the post-test period was also significantly significant (p=0.025). Although a positive difference was observed for relationships between providers working at the same facility, the difference between intervention and control groups was not statistically significant (p=0.110). The mean value of relationships between providers and clients was found to be slightly lower in Kono than in the comparison district, though the difference was not statistically significant (p=0.320).

### Association between change in coping skills, stress levels and change in relationships

To address objective 2, Table 2 presents the Pearson correlation coefficients between the changes in coping skills from the pre-test to the post-intervention, the overall post-test perceived stress levels, and the changes in relationships from the pre-test to the post-intervention for the 129 health workers interviewed in the intervention district. An increase in coping skills was associated with a reduction in stress levels and was statistically significant (-0.197, p<0.05), though the association was rather weak. The association was stronger for communications skills (-0.237, p<0.01), weaker for social connectedness (-0.177, p<0.05), and virtually non-existent for self-care skills. Lower post-test stress levels were associated with sharper increases in relationships. The three observed linear relationships, which were all statistically significant, appear rather weak for the relationships with co-workers (-0.202, p<0.05), for the relationships with patients (-0.191, p<0.05), and for the overall relationships variable (0.223, p<0.05).

**Table 2:** Pearson correlation coefficients between change in coping skills, stress levels, and change in relationships

	Overall stress level (N=129)	Change in Relationships		
		
		With co-workers (N=129)	With patients (N=129)	Overall (N=129)
Change in communication skills	-.237^**^	.281^**^	.388^**^	.408^**^

Change in self-care skills	-0.057	.184^*^	.242^**^	.282^**^

Change in social connectedness	-.177^*^	.335^**^	.381^**^	.465^**^

Change in overall coping skills	-.197^*^	.330^**^	.415^**^	.475^**^

Overall stress level	NA	-.202^*^	-.191^*^	-.223^*^

^**^p < 0.01

^*^p <0.05

The associations between changes in coping skills and changes in relationships were strong (+0.475, p<0.01). The correlation of change in overall coping skills was stronger with a change in relationships between providers and patients (+0.415, p<0.01), than with a change in relationships between co-workers (+0.330, p<0.01). Among the three domains of coping techniques, social connectedness and communication skills had the strongest correlations with relationships with co-workers (+0.335 and +0.281, respectively), and with relationships with patients (+0.381 and +0.388, respectively).

### In-depth insights on associations between coping skills and relationships

The main themes that emerged from the qualitative analysis of health worker feedback were perspective-taking and awareness, empathy, mutual accountability, and striking a work-life balance. For clients, the qualitative analysis revealed a primary concern with health worker behavior as an impediment to quality care. The following results address objective 3. Most health workers interviewed reported learning about and using coping and stress management skills that were completely unknown to them prior to the project. In their narratives, some respondents reported that they became more aware of compassionate client care techniques and acknowledged their importance both for improving quality of care and for improving their personal quality of life.

Health workers interviewed in the project district identified client health-seeking behavior patterns as one of their main causes of stress. For example, respondents described being “interrupted” by patients needing urgent care during times when they were not working. One interviewee said, “People in this community do not have respect for medical [providers]. They can even enter my room [of my home] without knocking the door [at odd hours].” Other health workers spoke about client delays in seeking care, not following medical advice and a perceived lack of gratitude as additional sources of frustration related to client behaviors. Perspective-taking, the cognitive or intellectual understanding of another person’s experience, was commonly reported as a useful skill in improving provider-client relationships during similar instances [18]. Many health workers reported that being able to see a problem from another person’s viewpoint is a skill they gained from the intervention, and which helped them cope with situations that presented stressors both for themselves and for their clients. One health worker made the following comment when asked how he would handle a previous altercation with a patient now that he had completed the training:

“I don’t think I will go out of my temper because I have now known that maybe the woman has a stress or a problem that is bothering her from her home, so I will talk to her nicely and try to know her problem and see how best I can help her.”

By looking beyond the situation at hand and taking stock of the potential broader issues causing the conflict from the client’s perspective, such as stress related to limited health resources, the respondent found an effective coping mechanism. The same health worker also discussed how it could be difficult to call patients because many were speaking loudly in the waiting area. The way he learned to cope with this stressor was to approach them and ask them to lower their voices as “others were struggling” whereas before the training he shouted at patients. He said, “I know that with time they will change through the way I am talking to them.”

Empathy, a more emotional than cognitive response, was another effective stress management tool reported by health workers to improve their interactions with clients [18]. One health worker explained:

‘‘I am very much patient with people which I did not use to have. I always put myself in people’s position, thinking that if I’m the one that was in that situation [how would] it have been? So because of that [I] will know how to talk to them in that cool manner.’’

Health workers were able to reflect on the change in their own behaviors and attitudes toward their clients after the counseling and training they received. One health worker remarked: “The project was able to change…the harsh way I use[d] to talk to patients. After the training I changed…When they come to the clinic…I don’t shout at them.”

Apart from provider-client interactions, many health workers also described positive impacts of the intervention on relationships with their colleagues. Respondents reported that they were better able to work as a team, gained a better understanding of mutual accountability, had increased empathy for one another, and generally had “amicable and cordial” relationships with co-workers after the intervention. One respondent commented:

‘‘You know that this work is not a single man’s work. We need to work as a team; you alone cannot do all the work...Before that program came, my co-workers [and I] were not in good terms but now I can see reasons, if you have done a bad job… I will show you where you do not understand.’’

Fostering solidarity among colleagues helped health workers channel frustration into more productive actions such as mutual training to prevent stress-causing mistakes in the future.

An additional finding from the qualitative analysis was the importance of striking a work-life balance and improving self-care for health workers. One respondent described the difficulties with self-care:

“Like for most of us the health workers, whenever we see patient in the morning packed in the hospital, we will hardly take our breakfast. You will keep on walking and by the time you will want to eat you will have no appetite to eat. I’m a human being I can also fall sick but it is very hard for me to treat myself.”

She also reported that she would treat other people’s children before treating her own and blamed this practice for the death of her son. From the training she learned that “you should not do that. Before solving another man’s problem, you should solve your own first.” By doing so, this respondent and others felt they were more capable of doing their jobs well because they were healthier, both physically and emotionally.

During all client FGDs in Kono, clients prioritized issues related to relationships between themselves and health workers as their main health service concerns. Clients mentioned absenteeism of health workers’ behavioral issues as major impediments to positive interactions. Most FGD participants did not report any changes or improvements in health workers’ behaviors and their interactions with them; those who did were hesitant, as they did not know if the changes were going to be consistent.

## Discussion

Overall, the results demonstrate that the HHWC intervention had a positive effect on coping skills, stress levels and provider-provider and provider-client relationships. Furthermore, linear relationships were observed between changes over time in coping skills, stress levels, and provider-provider and provider-client relationships; thus supporting the hypothesis proposed by the project’s theory of change.

While it is difficult to change some factors in low resource settings such as drug stock-outs, lack of training and poor infrastructure, providing existing health workers with coping and stress management techniques to persevere within these difficult environments and manage their emotional health can potentially have an impact on health service delivery and quality of care [14]. Resilience is one such tool, and is particularly important in remote areas and in countries like Sierra Leone where health worker shortages are dire, job satisfaction and motivation are poor, financial incentives are lacking and bottlenecks to quality care are numerous. Resilient individuals are better able to handle stress constructively, behave well in social situations, empathize with others, realize and appreciate their own self-worth and purpose, organize their workload and maintain a more positive attitude about their job. All of these benefits, in turn, lead to improved interactions with social and professional networks [19,20]. The improvements seen in health workers’ abilities to apply coping skills within the HHWC intervention suggests this psychosocial approach is a promising methodology for health worker resilience building within the context of Sierra Leone.

The intervention resulted in improvements in overall relationships. More specifically, health workers reported that their relationships with clients and with their colleagues improved. Positive relationships among co-workers have been linked to improved motivation whereas poor relationships have been associated with higher staff turnover, dissatisfaction (from both staff and clients) and poor health care delivery [2,10]. Qualitative data provided insight into the recognition by health workers of the importance of skills pertaining to relationship building with clients such as perspective-taking and empathy. Poor interactions and relationships between clients and providers have been shown to reduce perceptions of quality and use of services [21]. Because perceived poor quality of care and patient dissatisfaction can lead to underutilization of services, end-user experiences are critical to understanding provision of care dimensions and how interactions can affect care-seeking patterns [22]. Research shows that patient satisfaction with health services is associated with provider empathy [23] while health workers’ job satisfaction and retention are impacted by relationships with and appreciation by clients [2]. Clients did not report improved provider-client relationships during FGDs; most interventions at the health worker level trickle down to users very slowly.

The HHWC intervention can be utilized to increase skills aimed at enhancing social connectedness and communication, the two domains of coping techniques found to have the strongest interaction with changes in relationships with clients and co-workers. These two techniques inherently involve qualities and situations that would allow for better relationship building. Poor patient-provider communication, including effects of harsh, short-tempered language, or busy providers, has been linked with poor patient-provider relationships [21-23]. This further illuminates the need to explore a longer monitoring period to allow for behavioral effects of the intervention to permeate to the community.

The increase in overall coping skills of health workers in the course of the intervention was significantly associated with lower stress levels. Among coping skills, the results showed that the associations between improvements in communication skills and social connectedness on the one hand and lower stress levels on the other hand were stronger than those between the change in self-care skills and stress levels. As such, the HHWC intervention can serve as a working model to specifically address stress and burnout, for which strategies have not been well documented, through improvements in these two coping mechanisms [17]. Addressing burnout, the long-term stress leading to professional demotivation and dissatisfaction, is essential among frontline health workers in order to improve the quality of care they are able to deliver [17]. Though not realized in the HHWC intervention, promoting and teaching self-care to health workers has been identified as a strategy to prevent burnout [17]. Since personal behaviors are hard to change and often require support from health management and the community, it is possible that further focus on self-care could strengthen the HHWC intervention and outcomes.

### Limitations

The lack of a baseline constitutes the main limitation of this study. While efforts were made to fill this gap through a retrospective assessment in the intervention area carried out after the intervention was delivered, biases associated with retrospective questioning and with surveys of satisfaction and motivation may have affected the results. Despite this limitation, the strong patterns of associations observed in the changes from the retrospective pre-test to the post-test periods suggest the effect of these biases may be small. Budgetary and logistical constraints did not allow us to better capture clients’ perspectives, which could be done through exit interviews at the health facility.

## Conclusions

This study exposes how a psychosocial intervention can impact the well-being and stress levels of health providers and the relationships they have with colleagues and clients in resource-poor settings, a strategy with great potential but little uptake. It is vital that health workers develop the capacity to recognize stressful situations that adversely affect them and not ignore the warning signs, but rather take initiative to address them at an early stage using learned coping skills. As was done through the HHWC intervention, health facility management has the responsibility to support the development of coping skills within low-resourced health settings. Our findings indicate that HHWC is a model and tool that could be used to build resilience, specifically by improving coping skills and stress management of health workers and the interaction between the two; and to improve the delivery of care through better teamwork and interactions among co-workers and improved communication and relationships with clients. This approach could also be instrumental in preventing burnout and enhancing the retention of health workers in LMICs, complementing other strategies such as incentives.

A rollout of the HHWC approach alongside health system strengthening could have major positive implications for improving health and survival through increased health service access and delivery. The HHWC intervention presents a low-cost and relatively rapid method to potentially improve maternal, newborn and child health outcomes, as this is a major focus of frontline health workers within many low resource settings. Integrating psychosocial counseling and training interventions into health worker pre-service and in-service curricula would allow the positive effects seen in Kono to be scaled up across Sierra Leone and beyond. The HHWC model could also be applied in the rehabilitation and recovery of a humanitarian crisis such as the Ebola virus outbreak in Sierra Leone, an ultimate test of the resilience of health workers given the breakdown of health systems.

## Competing interests

The authors declare that they have no completing interests.

## Authors’ contributions

JCF and JD carried out the analysis. LV wrote the manuscript. KW, JD and JCF contributed to the drafting and critical revision of the manuscript. All authors read and approved the final manuscript.

## Supplementary Material

Additional file 1 Click here for file

## Declarations

The publication costs for this article were funded by the Innovations for Maternal, Newborn & Child Health, an initiative funded by the Bill & Melinda Gates Foundation.

This article has been published as part of *BMC Health Services Research* Volume 15 Supplement 1, 2015: Improved access to maternal, newborn and child health services: strengthening human resources for health. The full contents of the supplement are available online at http://www.biomedcentral.com/bmchealthservres/supplements/15/S1.
