# Supplementary material for: Psychosocial support and resilience building among health workers in Sierra Leone: interrelations between coping skills, stress levels, and interpersonal relationships
Source: BMC Health Serv Res. 2015 Jun 8;15(Suppl 1):S3. doi: 10.1186/1472-6963-15-S1-S3 (PMC4464206; doi:10.1186/1472-6963-15-S1-S3)
Supplement: Additional file 1 [file 1472-6963-15-S1-S3-S1.docx]

**Annex 1:** Selected sections of the survey questionnaire

**1. Coping techniques**

| **Would you say…** | Never | Not often | Often | Always |
| --- | --- | --- | --- | --- |
|  | **1** | **2** | **3** | **4** |
| ***Communication skills*** | | | | |
| 1. You are able to stay focused on a conversation even though you may be feeling bad about the topic/issue |  |  |  |  |
| 1. You are able to talk to people nicely even though you may be angry |  |  |  |  |
| 1. You are able to say ‘sorry’ even though it may be hard |  |  |  |  |
| 1. You are able to respect someone else’s view point |  |  |  |  |
| ***Self-care skills*** | | | | |
| 1. You are getting enough sleep |  |  |  |  |
| f. You are eating regular, healthy meals |  |  |  |  |
| 1. You seek health care when you need it |  |  |  |  |
| 1. You are able to take time off work to relax and do other things |  |  |  |  |
| 1. You able to say “no” to more work or other activities when you are very busy |  |  |  |  |
| ***Social connectedness*** | | | | |
| 1. You meet people in your community regularly to talk and relax |  |  |  |  |
| k. You meditate or pray^[[1]](#footnote-1)^ |  |  |  |  |
| 1. You find ways to relax (e.g. sing, dance, listen to music)^[[2]](#footnote-2)^ |  |  |  |  |
| 1. You can confidently talk about things that you feel are important for your happiness in life^[[3]](#footnote-3)^ |  |  |  |  |
| 1. You able to spend time by yourself to do things that make you happy^[[4]](#footnote-4)^ |  |  |  |  |
| 1. You feel you have friends and family who support you |  |  |  |  |

**2. Perceived Stress**

| **Have you…** | Never | Not often | Often | Always |
| --- | --- | --- | --- | --- |
|  | **1** | **2** | **3** | **4** |
| 1. Been upset because of something that happened unexpectedly |  |  |  |  |
| 1. Felt that you were unable to control important things |  |  |  |  |
| 1. Felt nervous and "stressed" |  |  |  |  |
| 1. Felt confident about your ability to handle your personal problems |  |  |  |  |
| 1. Felt confident about your ability to handle any work problems |  |  |  |  |
| 1. Felt that things were working out well |  |  |  |  |
| 1. Found that you could not cope with all the jobs that you had to do |  |  |  |  |
| 1. Been able to control irritations |  |  |  |  |
| 1. Felt that you were able to handle things that came your way |  |  |  |  |
| 1. Been angry because of things that were outside of your control |  |  |  |  |
| 1. Felt difficulties were piling up so high that you could not overcome them |  |  |  |  |

**3. Relationships**

| **What would you say about …** | Good | Some-what good | Not good | Bad | No relation-ship |
| --- | --- | --- | --- | --- | --- |
|  | **1** | **2** | **3** | **4** | **5** |
| 1. Your relationship with your supervisor |  |  |  |  |  |
| 1. Your relationships with your co-workers at your health facility |  |  |  |  |  |
| 1. Your relationship with co-workers from other health facilities nearby |  |  |  |  |  |
| 1. Your relationship with TBAs working in your community |  |  |  |  |  |
| 1. Your relationship with patients who come to the facility |  |  |  |  |  |

1. Prayer was thought of first and foremost as a community activity in the study setting since places like churches and mosques also serve as places for socialization. For this reason, the variable was recorded as part of the category on social connectedness. [↑](#footnote-ref-1)
2. The rationale for recording this variable in the social connectedness category is that dancing and singing were seen to be largely group activities. [↑](#footnote-ref-2)
3. The focus is on talking about issues that are important to happiness. This variable was trying to capture whether the respondent had trusted channels for discussing confident matters. For this reason, the variable was included in the category on social connectedness. [↑](#footnote-ref-3)
4. In the Sierra Leone context, this variable was understood as something that the participant would initiate him/herself and these could be activities such as going to see friends and relatives. For this reason, the variable was included in the category on social connectedness. [↑](#footnote-ref-4)
